# Artificial intelligence for radiographic imaging detection of caries lesions: a systematic review

**DOI:** 10.1186/s12903-024-04046-7

**Published:** 2024-02-24

**Authors:** Domenico Albano, Vanessa Galiano, Mariachiara Basile, Filippo Di Luca, Salvatore Gitto, Carmelo Messina, Maria Grazia Cagetti, Massimo Del Fabbro, Gianluca Martino Tartaglia, Luca Maria Sconfienza

**Affiliations:** 1 IRCCS Istituto Ortopedico Galeazzi, Milan, Italy; 2 Department of Biomedical, Surgical and Dental Sciences, University of Milan, Milan, Italy; 3 School of Dentistry, University of Milano, Milan, Italy; 4 Postgraduate School of Diagnostic and Interventional Radiology, University of Milan, Milan, Italy; 5 Department of Biomedical Sciences for Health, University of Milan, Milan, Italy; 6 Ospedale Maggiore Policlinico, UOC Maxillo-Facial Surgery and Dentistry Fondazione IRCCS Cà Granda, Milan, Italy

**Keywords:** Artificial intelligence, Caries lesion, Radiographic imaging, Detection, Diagnosis

## Abstract

**Background:**

The aim of this systematic review is to evaluate the diagnostic performance of Artificial Intelligence (AI) models designed for the detection of caries lesion (CL).

**Materials and methods:**

An electronic literature search was conducted on PubMed, Web of Science, SCOPUS, LILACS and Embase databases for retrospective, prospective and cross-sectional studies published until January 2023, using the following keywords: artificial intelligence (AI), machine learning (ML), deep learning (DL), artificial neural networks (ANN), convolutional neural networks (CNN), deep convolutional neural networks (DCNN), radiology, detection, diagnosis and dental caries (DC). The quality assessment was performed using the guidelines of QUADAS-2.

**Results:**

Twenty articles that met the selection criteria were evaluated. Five studies were performed on periapical radiographs, nine on bitewings, and six on orthopantomography. The number of imaging examinations included ranged from 15 to 2900. Four studies investigated ANN models, fifteen CNN models, and two DCNN models. Twelve were retrospective studies, six cross-sectional and two prospective. The following diagnostic performance was achieved in detecting CL: sensitivity from 0.44 to 0.86, specificity from 0.85 to 0.98, precision from 0.50 to 0.94, PPV (Positive Predictive Value) 0.86, NPV (Negative Predictive Value) 0.95, accuracy from 0.73 to 0.98, area under the curve (AUC) from 0.84 to 0.98, intersection over union of 0.3–0.4 and 0.78, Dice coefficient 0.66 and 0.88, F1-score from 0.64 to 0.92. According to the QUADAS-2 evaluation, most studies exhibited a low risk of bias.

**Conclusion:**

AI-based models have demonstrated good diagnostic performance, potentially being an important aid in CL detection. Some limitations of these studies are related to the size and heterogeneity of the datasets. Future studies need to rely on comparable, large, and clinically meaningful datasets.

**Protocol:**

PROSPERO identifier: CRD42023470708

**Supplementary Information:**

The online version contains supplementary material available at 10.1186/s12903-024-04046-7.

## Background

Dental caries is a chronic disease that culminates in dental decay. It stems from a complex interplay between acids produced by bacteria adhering to the teeth and fermentable carbohydrates. As time progresses, the acids in dental plaque can demineralize enamel and dentin, giving rise to white spot lesions, which, if demineralization persists, may progress into cavities. It is a multifactorial disease that, in many cases, goes undiagnosed, particularly when it is interproximal or in the early stage. Risk factors include high numbers of cariogenic bacteria, high-frequency sugar consumption, inadequate salivary flow, insufficient fluoride exposure, poor oral hygiene, and poverty [1].

Early and accurate detection of a caries lesion (CL) can lead to better preventive and conservative measures, thereby reducing healthcare costs [2]. Clinical examination, in combination with radiographic evaluation, is the routine diagnostic approach. However, previous studies have shown the substantial variability of its reliability and accuracy, influenced mainly by the degree of experience of dentists. Sensitivity can vary between 0.19 and 0.92 for occlusal CL and 0.39–0.94 for interproximal CL [3].

Artificial intelligence (AI) is a discipline in computer science concerned with creating ‘machines’ that can mimic the cognitive capabilities of human intelligence [4–6].

Recent implementations of AI for imaging rely on deep learning (DL), a subfield of machine learning [7]. DL diverged from previous machine learning (ML) methods by replacing features engineered by humans with high-capacity neural networks trained on extensive datasets, allowing for automated feature extraction. To date, the most effective models for image analysis are convolutional neural networks (CNNs). CNNs consist of many layers that transform their input using convolution filters of a limited extent [8].

Since the advent of DL, it has been suggested for various applications in the field of oral and dental health, such as tooth classification, detection and segmentation [9, 10], endodontic treatment and diagnosis [11], periodontal problem tooth [12] and oral lesion pathology detection [13].

In particular, this study focuses on how these new methods can overcome the constraints of clinical and radiographic imaging diagnosis in the detection of CLs. The development of software that enables the automatic detection of CL seems to improve diagnostic accuracy, easing the observer’s workload and making AI a powerful tool for clinical practice. For example, a DL model, after a period of training and validating, has been shown to be able to detect CLs with sensitivity, specificity, and accuracy even higher than 0.80 [14]. Therefore, the question is, could AI somehow “replace” the dentist or radiologist in detecting CLs? According to data provided by the Food & Drug Administration this is partly possible. For instance, Videa Caries Assist, a recently commercialized AI model, leads to a 0.43 decrease in undetected CLs and a 0.15 reduction of misdiagnoses, regardless of the dentist’s experience [15]. These are non-negligible data, which consequently reflect in earlier, less invasive, cheaper, and less painful treatments for patients. Further, Mohammad-Rahimi et al. [16], evaluating the economic impact of AI-based models, stated that the application of AI for CLs detection seems justified with the costs incurred to implement it. The new opportunities offered by AI have been acting as a driving force for research in this setting. More and more startups are showing interest in this field and working to revolutionize dental imaging. Indeed, an increasing number of studies investigated caries detection by means of deep learning reporting promising accuracy and reliability.

Hence, the purpose of this systematic review is to investigate the diagnostic performance of AI-based modalities designed for the detection of CL.

## Materials and methods

### Study design

This systematic review of the literature was conducted according to the Preferred Reporting Items for Systematic Reviews and Meta-Analyses (PRISMA) guidelines [17]. The protocol was registered in the International Prospective Register of Systematic Reviews database (PROSPERO identifier: CRD42023470708). Full PRISMA checklist can be found in Additional file 1.

### Data source

A literature search was conducted on the PubMed, Web of Science, SCOPUS, LILACS and Embase databases for articles published from inception until January 2023. An investigation was also carried out on gray literature databases such as OpenGrey and WONDER.

The search was conducted with the following combination of MeSH terms and keywords using Boolean operators: (detection OR diagnosis OR radiological imaging) AND (dental caries OR caries lesion OR decay OR white spot OR cavity) AND (artificial intelligence OR AI OR machine learning OR deep learning OR artificial neural networks OR convolutional neural networks OR deep convolutional neural networks).

### Data selection

All English-language studies were first screened by title and abstract. Then, the full text of the eligible studies was retrieved for further review. The bibliography of identified publications was checked to assess the possible inclusion of additional publications. The bibliographic search and study selection were performed by one reviewer (with 2 years of experience) and checked by a senior researcher with 10 years of experience.

### Eligibility criteria

The inclusion criteria were: (i) original research studies concerning diagnostic performance AI-based models in the detection of CL, (ii) articles reporting the datasets used for training/validation and testing of the model, (iii) the type of study design did not limit inclusion, (iv) studies involving human participants, (v) English language, (vi) approval of the local ethics committee and informed consent of each patient (or a waiver for it). The exclusion criteria were: (i) studies that reported insufficient data, (ii) case reports and case series involving less than 10 images, narrative reviews, guidelines, consensus statements, editorials, letters, comments, or conference abstracts. We considered DL-based models for detection of CL based on dental imaging as index test, with radiographs assessment performed by expert dentists as reference test.

### Data extraction and meta-analysis

Data on the following parameters were extracted and analyzed:

(I) study characteristics: authorship, year of publication and study design;
(II) number and type of radiographic examinations (the number of training images was not included);
(III) diagnostic performance: sensitivity, specificity, precision, positive predictive value (PPV), negative predictive value (NPV), accuracy, area under the curve (AUC), false positive, intersection over union (IoU), Dice coefficient, F1-score;
(IV) type of AI algorithms used: deep neural network (DCNN), convolutional neural network (CNN), artificial neural networks (ANNs);
(V) main results.

Studies included were critically analyzed based on the guidelines of quality assessment and diagnostic accuracy tool (QUADAS-2) [18]. Studies characteristics were resumed in tables. If at least two works presented the same outcome, data from both studies were pooled in a meta-analysis using a random effect model [19–22]. We used the mean difference (MD) with 95% confidence interval (CI) when the same measurement method was applied. Statistical heterogeneity was evaluated through the I² statistic. We used Review Manager (RevMan) software version 5.3 for this meta-analysis.

## Results

### Study characteristics

**Fig. 1:**
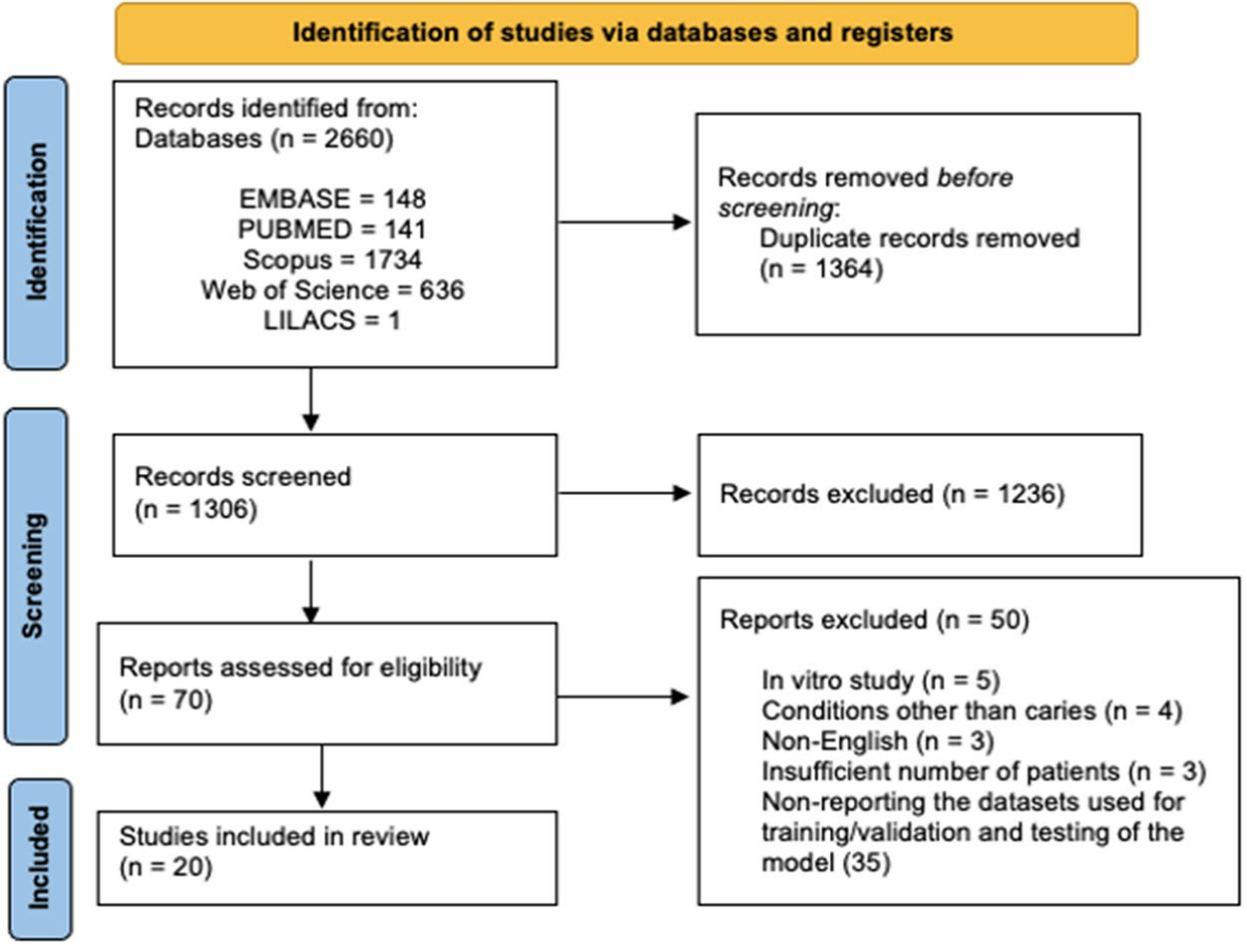
Flow chart of articles’ screening and selection

Initial literature research allowed to retrieve 2660 records, of which 1364 were removed as duplicates. After title and abstract screening, 1236 were excluded due to lack of pertinence. The remaining 70 articles were evaluated in the full-text format based on the eligibility criteria. Finally, 20 articles were included (Fig. 1):

(I) Five articles were performed on periapical radiographs, nine on bitewings and six on orthopantomography. The number of radiographs used in each study to build the model ranged from 15 to 2900, for a total of 6346 images analyzed for CLs detection.
(II) Four studies investigated ANN models, fourteen CNN models and two DCNN model. So, 70% of the included studies have used CNN-based models.
(III) Twelve studies were retrospective, six were cross-sectional and two prospective.

**Table 1:** Details of the studies that used AI-based models for diagnosis of CLs

Authors	Year	Objective of the Study	Design	X-Ray Examinations			
				Periapical Radiographs	Bitewings	Orthopantomography	Tot
Devito et al. [23]	2008	Detection of CLs	Retrospective cohort	0	160	0	160
Lee et al. [24]	2018	Detection of CLs	Retrospective cohort	600	0	0	600
Choi et al. [25]	2018	Detection of CLs	Retrospective cohort	475	0	0	475
Cantu et al. [26]	2020	Detection of CLs	Retrospective cohort	0	141	0	141
Geetha et al. [27]	2020	Detection of CLs	Cross-sectional	145	0	0	145
Chen et al. [28]	2021	Detection of CLs/PD	Retrospective cohort	2900	0	0	2900
Devlin et al. [29]	2021	Detection of CLs	Randomized control trial	0	24	0	24
Bayrakdar et al. [30]	2021	Detection of CLs	Retrospective cohort	0	53	0	53
Lian et al. [31]	2021	Detection of CLs	Cross-sectional	0	0	89	89
Moran et al. [32]	2021	Detection of CLs	Cross-sectional	0	45	0	45
Mertens S et al. [33]	2021	Detection of CLs	Randomized control trial	0	20	0	20
Vinayahalingam et al. [34]	2021	Detection of CLs	Retrospective cohort	0	0	100	100
Lee et al. [35]	2021	Detection of CLs	Cross-sectional	0	50	0	50
Hur et al. [36]	2021	Detection of CLs	Retrospective cohort	0	0	792	792
De Araujo Faria et al. [37]	2021	Detection of CLs	Retrospective cohort	0	0	15	15
Mao et al. [38]	2021	Detection of CLs	Cross-sectional	0	83	0	83
Bayraktar et al. [39]	2022	Detection of CLs	Cross-sectional	0	200	0	200
Zhu et al. [40]	2022	Detection of CLs	Retrospective cohort	0	0	124	124
Zadrozny et al. [41]	2022	Detection of CLs	Retrospective cohort	0	0	30	30
Shihao Li et al. [14]	2022	Detection of CLs/PD	Retrospective cohort	300	0	0	300

CLs = Caries Lesions, PD = Periodontal Disease

Data were retrieved and included into Tables 1 and 2. AI has been applied for detection and classification of CLs. Not all the studies detailed how caries was defined, and not all detailed the modality of carious lesion detection. Meta-analysis could not be performed due to the lack of sufficient data and the heterogeneity between the studies, specifically in the software (different neural network), datasets used to evaluate the performance of AI models, and outcome metrics. Therefore, descriptive data were presented based on the application of the AI models for which they were designed.

#### Diagnostic performance (table 2)

(I) Eleven studies analyzed sensitivity, obtaining the following outcomes: a range from 0.44 to 0.86 (mean ± standard deviation [SD] of 0.75 ± 0.13, median of 0.75);
(II) Five studies analyzed specificity, obtaining the following outcomes: a range from 0.83 to 0.98 (mean ± SD of 0.90 ± 0.07, median of 0.88);
(III) Four studies analyzed precision, obtaining the following outcomes: a range from 0.50 to 0.94 (mean ± SD of 0.73 ± 0.17, median of 0.72);
(IV) Ten studies analyzed accuracy, obtaining the following outcomes: a range from 0.73 to 0.98 (mean ± SD of 0.89 ± 0.08, median of 0.91);
(V) Eight studies analyzed AUC, obtaining the following outcomes: a range from 0.84 to 0.98 (mean ± SD of 0.92 ± 0.04, median of 0.88);
(VI) Six studies analyzed F1-score, obtaining the following outcomes: a range from 0.64 to 0.92 (mean ± SD of 0–80 ± 0.09, median of 0.83);
(VII) Few studies analyzed the rest of the diagnostic performance: only 2 studies analyzed IoU (0.3–0.4 and 0.78) and Dice coefficient (0.66 and 0.78); only one study analyzed PPV (0.86) and NPV (0.95).

**Table 2:** Diagnostic performance of different AI-based models of all selected studies

Authors	Year	Artificial intelligence			Diagnostic performance/Outcome Metrics											Index test
		DCNN	CNN	ANN	Sens	Spec	Prec (%)	PPV(%)	NPV (%)	Acc (%)	AUC	FP (%)	IoU	Dice	F1	
Devito et al. [23]	2008	N	N	Y							0.88					
Lee et al. [24]	2018	Y	N	N						82	0.84					GoogLeNet Inception-v3
Choi et al. [25]	2018	N	Y	N											0.74	
Cantu et al. [26]	2020	N	Y	N	0.75	0.83				80						U-Net
Geetha et al. [27]	2020	N	N	Y						97.10	0.98	2.80				BPNN
Chen et al. [28]	2021	N	Y	N	0.55		55						0.35			Faster R
Devlin et al. [29]	2021	N	Y	N	0.75	0.85										AssistDent
Bayrakdar et al. [30]	2021	N	Y	N	0.84		81								0.84	VGG-16, U-Net
Lian et al. [31]	2021	N	Y	N	0.82					98.6			0.78	0.63		nnU-Net
Moran et al. [32]	2021	N	Y	N						73.3						ResNet, Inception
Mertens S et al. [33]	2021	N	Y	N	0.81						0.89					
Vinayahalingam et al. [34]	2021	N	Y	N	0.86	0.88				87	0.90				0.86	GoogLeNet Inception-v 3
Lee et al. [35]	2021	N	Y	N	0.65		63.29								0.64	U-Net
Hur et al. [36]	2021	N	N	Y							0.89					
De Araujo Faria et al. [3]	2021	N	N	Y						98.8	0.98					
Mao et al. [38]	2021	N	Y	N						90.3						Faster R
Bayraktar et al. (39)	2022	N	Y	N	0.72	0.98		86.58	95.64	94.59	0.87					YOLO
Zhu et al. [40]	2022	N	Y	N	0.86		94.09			93.61				0.93	0.92	CariesNet
Zadrozny et al. [41]	2022	N	Y	N	0.44	0.98										Diagnocat
Shihao Li et al. [14]	2022	Y	N	N											0.82	ResNet

ANN = artificial neural networks, CNN = convolutional neural networks, DCNN = deep neural networks, Y = yes, N = no, Sens = sensitivity, Spec = specificity, PPV = positive predictive value, NPV = negative predictive value, Acc = accuracy, AUC = Area Under the Curve, FP = False Positive, IoU = Intersection over Union, Dice = Dice coefficient, F1 = F1-score, measure of test’s accuracy

### Risk of bias assessment and applicability concerns

**Fig. 2:**
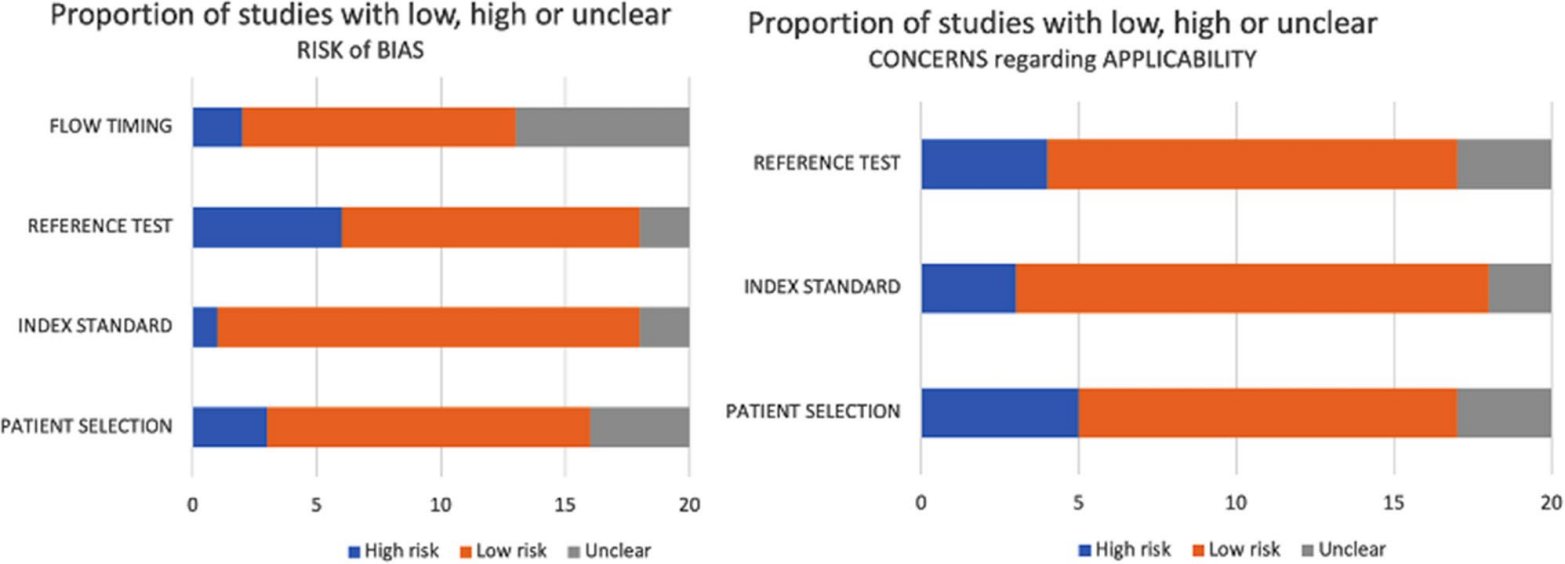
QUADAS-2 assessment of the individual risk of bias domains and applicability concerns

The quality assessment of the 20 studies included in this systematic review was performed using the guidelines of QUADAS-2 [18], consisting of 4 key domains: patient selection, index test, reference flow and timing. According to the QUADAS-2 evaluation of risk and applicability most studies included in this work exhibited a low risk of bias, with only a few displaying a high risk. (Additional file 2) (Fig. 2).

### Results of included studies

One of the earliest published studies evaluating the diagnostic performance of AI-based software was conducted by Devito et al. [23] in 2008 and highlighted the potential use of the neural network model showing a diagnostic improvement of 0.39 using ANN, AUC was 0.88. In 2018, Lee et al. [24] demonstrated that the AI CapsNet (CNN-based) model for CL detection in periapical radiographs showed an accuracy of 0.89, 0.88 and 0.82 and an AUC of 0.91, 0.89 and 0.84, for premolars, molars and both premolar and molar models, respectively. In the same year, Choi et al. [25], suggested that the system using a CNN and crown extraction is superior to the system using a naïve CNN (F1-score 0.74), based on experiments conducted on various periapical images.

Cantu et al. [26] in 2020 proposed a deep neural network to detect caries lesions at an early stage on bitewing radiographs, which showed an accuracy of 0.80, significantly higher than the dentist’s one. The main strength of this study was the huge amount of data used in training and testing. These results were similar to those of another study conducted by Lee et al. [35] in 2021 on a CNN (U-Net) model, which demonstrated an accuracy of 0.63 and a F1-score of 0.64. However, the limitation of the latter study is related to the low number of data used, since only 50 bitewing radiographs were used for CNN performance evaluation. Geetha et al. [27] reported excellent performance of the AI-based model proposed, with an accuracy of 0.97 and a false positives (FP) rate of 0.02, despite the need to improve the system for classification of caries depth and datasets quality and quantity. Chen et al. [28] demonstrated that CNNs can detect different pathologies on dental periapical radiographs, both CLs and periodontal disease, more easily if they are severe. Lesions were generally detected with precision value of 0.5–0.6. The authors also concluded that it would be better to train CNNs with a customized strategy for each pathology. Devlin et al. [29] concluded that their AI-based software significantly improved (0.71 sensitivity) the ability of dentists to detect CLs and, therefore, can be considered a tool to support preventive dentistry. The models studied in 2021 by Bayrakdar et al. [30] demonstrated superior performance compared to experienced specialists in CLs detection and may be useful for dentists in clinical decision-making, despite the limited datasets including just 53 bitewing radiographs.

A more recent article [39] published a year later and based on a larger dataset, demonstrated the excellent performance of the CNN model in diagnosing interproximal dental caries, with sensitivity of 0.72, specificity of 0.98, PPV of 0.86, NPV of 0.95, accuracy of 0.94 and AUC of 0.87. Nevertheless, the model could not classify DC according to their location in the enamel and/or dentin, which was one of the main limitations of this study. According to Lian et al. [31] the performance of deep learning methods in detecting and classifying CLs on panoramic radiographs was comparable to that of expert dentists, with Sørensen similarity index values of 0.66 and accuracy of 0.98. However, the dental panoramic radiographs used in this study were obtained from a single orthopantomograph, therefore, performance may vary using OPG from different manufacturers and Institutions. The CNN-based model studied by Moran et al. [32] showed promising results compared to the reference model (accuracy 0.73) suggesting potential application of the proposed method a supplementary resource for the dentist in the evaluation of bitewing images. Mertens et al. [33] focused on a CNN model that showed an AUC of 0.89 and a sensitivity of 0.81, with significant better results compared to five experienced dentists. The primary merit of this study stemmed from its utilization of a randomized controlled trial design. On the other hand, the main drawback was the limited sample of data sets available, which included just 20 bitewing radiographs. Vinayahalingam et al. [34] evaluated a method that achieved accuracy of 0.87, sensitivity of 0.86, specificity of 0.88 and AUC of 0.90 for the classification of CLs of the third molars. The model analyzed by Hur et al. [36] also showed remarkable performance in detecting caries in mandibular third molars (M3M) and mandibular second molars (DCM2M), with accuracy of 0.63 and F1-score of 0.64. These prediction models (ANN based) could be used to detect patients at a high risk of developing DCM2M and ultimately contribute to caries prevention and treatment decision-making for impacted M3Ms. The study conducted by De Araujo Faria et al. [37] reported excellent CLs detection accuracy (0.98) with an AUC of 0.98. For prediction, it showed an accuracy of 0.99 and an AUC of 0.98. These results were achieved including only 15 orthopantomography examinations. Another study [38] conducted by Mao et al. used a CNN-based model, namely AlexNet, which showed 0.90 accuracy in detecting DC, significantly high compared to other models. This study has the potential to enhance classification accuracy and, consequently, decrease the time required for clinical procedures, allowing dentists to concentrate more on treatment planning and clinical operations. The model studied by Zhu et al. [40], in 2022, also showed excellent performance, with an average Dice coefficient of 0.93, an accuracy of 0.93, a F1-score of 0.92 and precision of 0.94. The large number of data (124 orthopantomography examinations) used to train and validate the model was a strength of this study. Zadrozny et al. [41] concluded that their AI model based on CNN could be useful for an initial assessment of orthopantomography (sensitivity 0.44; specificity 0.98) as an aid for dentists in imaging interpretation. Li et al. [14] also affirmed that CLs models possess the capability to automatically identify CLs and periodontal disease with greater sensitivity and specificity than unassisted decision-making evaluation (F1- score 0.82).

## Discussion

In this systematic review, we have assessed studies that used AI methods to detect CL based on dental images. With the advancing technology of AI, an increasing number of articles have investigated the diagnostic performance of AI-based models for CL detection, particularly between 2018 and 2022. However, the overall quality of the included studies was found to be limited, emphasizing the urgent need for more high-quality research in this specific area. Nevertheless, it’s worth noting that most articles included in this review reported good diagnostic performance of their algorithms.

In the field of DL, CNN is the most commonly employed algorithm [42]. The structure of CNNs was inspired by neurons in the human brain, similar to a conventional neural network. CNNs learn statistical patterns in images by repetitively analyzing pairs of images and image labels. Eventually, CNNs become proficient in evaluating previously unseen data [43]. Goodfellow et al. identified three key benefits of the CNN: equivalent representations, sparse interactions, and parameter sharing [44]. This data is reflected in our work, in which 70% of the included studies have chosen to adopt a CNN-based model recording a high level of accuracy. Various CNN architectures were used in this review, such as GoogLeNet Inception v3, U-Net, Faster R-CNN, ResNet. On the other hand, only 30% of considered articles have examined ANN-based or DCNN-based models. Anyway, the diagnostic accuracy seems to be comparable within the different subgroups.

Then, based on the included publications, transfer learning is an effective method for training datasets with a limited number of samples, enhancing overall model training efficiency. In addition, the utilization of suitable regularization methods can improve model performance.

With respect to dental images modalities, the studies included in our analysis used periapical radiographs, bitewings radiographs and orthopantomography for CLs detection. Bitewing radiographs have been shown to be the best diagnostic tool for the detection of interproximal dental caries [45]. In fact, nearly half of the studies in our analysis detected CLs in bitewing radiograph images. Nevertheless, the experience in clinical practice is the most influential factor. Compared with experienced examiners, low-experienced examiners are about four times as likely to make incorrect assessments when diagnosing proximal CL [46]. For this reason, an automated assistance system for dental radiography images may help to address these shortcomings by providing a reliable and stable diagnostic result, especially for less-experienced examiners.

AI has revolutionized dentistry in the last few years. Studies show that these AI-powered automated systems performed extremely well in various scenarios. Few authors found them to be more accurate than even dental specialists. For example, Bayrakdar et al. [30] demonstrated superior performance of the CNN algorithms under investigation, VGG-16 and U-Net, compared to experienced specialists in CLs detection. Similarly, the CNN-based models studied by Moran et al. [32] and Mertens et al. [33] reported significantly higher sensitivity and accuracy values compared to the reference test. As abovementioned, the model studied by Zhu et al. [40], also showed excellent performance using 124 orthopantomography examinations to train and validate the model. Although these outcomes do not make them better than clinicians, they do establish that AI may be useful for dentists in clinical decision-making. This is a remarkable benefit because it can help professionals to diagnose cases in the early stages, rendering best quality care to their patients.

With the growing number of AI products, it becomes essential for physicians to actively participate in selecting and applying these technologies. Instead of replacing dentists and radiologists, the integration of AI may assist them in streamlining workflow, enhancing diagnostic capabilities, and managing the rising workload [47].

Some limitations should be pointed out. We limited our literature search just to English papers. Then, we included studies with different design focused on different AI-types algorithms. However, we were not able to do a meta-analysis due to the low number and high heterogeneity of published studies on this topic. Hence, further studies are warranted to understand the potential role of AI-based models in CL imaging detection.

## Conclusions

In conclusion, AI-based models exhibit good diagnostic performance in detecting CLs using dental images. AI-based models can be efficient methods for reducing the workload of dentists and the time spent in clinical practice. Additionally, the various AI models come with their own set of pros and cons, so the selection of a model should be based on the specific task objectives and requirements. Future research should be designed to accurately represent the true performance of AI models. Optimizing the models architecture holds the potential to enhance the performance of CL detection in dental images, thereby improving diagnostic accuracy. Concerning the implications of AI, users must always critically evaluate the accuracy of these diagnostic support systems, the data underlying the trained models and its tests, as the ultimate decision-maker for any decision arising from the use of a diagnostic support system remains the user himself.

### Electronic supplementary material

Below is the link to the electronic supplementary material.

Supplementary Material 1: PRISMA checklist

Supplementary Material 2: QUADAS-2

## Data Availability

The datasets used and/or analyzed during the current study are available from the corresponding author on reasonable request.

## Abbreviations

AI: Artificial Intelligence
ANN: Artificial Neural Networks
AUC: Area Under the Curve
CL: caries lesion
CNN: Convolutional Neural Networks
DCNN: Deep Convolutional Neural Networks
DL: Deep Learning
FP: False Positive
IoU: Intersection over Union
ML: Machine Learning
NPV: Negative Predictive Value
PPV: Positive Predictive Value
SD: Standard Deviation
