# Supplementary material for: Artificial intelligence for radiographic imaging detection of caries lesions: a systematic review
Source: BMC Oral Health. 2024 Feb 24;24:274. doi: 10.1186/s12903-024-04046-7 (PMC10894487; doi:10.1186/s12903-024-04046-7)
Supplement: Supplementary file 2 — Supplementary Material 2: QUADAS-2 [file 12903_2024_4046_MOESM2_ESM.pdf]

# Additional file 2. QUADAS-2

| | RISK OF BIAS | | | | APPLICABILITY CONCERNS | | |
|---|---|---|---|---|---|---|---|
| Study | PATIENTS SELECTION | INDEX TEST | REFERENCE STANDARD | FLOW TIMING | PATIENTS SELECTION | INDEX TEST | REFERENCE STANDARD |
| Devito et al. | Unclear | Low risk | Low Risk | Low risk | High risk | Low risk | High risk |
| Lee et al. | Low risk | Low risk | Low risk | Unclear | Low risk | Low risk | Low risk |
| Choi et al. | Low risk | Unclear | High risk | High risk | Low risk | Unclear | High risk |
| Cantu et al. | Low risk | Low risk | High risk | Low risk | Low risk | High risk | Unclear |
| Geetha et al. | High risk | Low risk | Unclear | Low risk | Low risk | Low risk | Low risk |
| Chen et al. | Low risk | Low risk | Low risk | Unclear | Low risk | High risk | Low risk |
| Devlin et al. | Low risk | Low risk | Low risk | Low risk | Low risk | Low risk | Low risk |
| Bayrakdar et al. | Low risk | Low risk | Low risk | Low risk | Low risk | Low risk | Low risk |
| Lian et al. | Low risk | Low risk | High risk | Unclear | Low risk | Low risk | Low risk |
| Moran et al. | Unclear | Low risk | Low risk | Low risk | Unclear | Low risk | Low risk |
| Mertens S et al. | Unclear | Low risk | Low risk | Low risk | Unclear | Low risk | Low risk |
| Vinayahalingam et al. | High risk | Unclear | High risk | Unclear | High risk | Unclear | Unclear |
| Lee et al. | High risk | Low risk | High risk | Unclear | High risk | High risk | High risk |
| Hur et al. | Low risk | High risk | High risk | Unclear | High risk | Low risk | Low risk |
| De Araujo Faria et al. | Low risk | Low risk | Low risk | Low risk | Low risk | Low risk | Low risk |
| Mao et al. | Unclear | Low risk | Unclear | Unclear | Unclear | Low risk | Low risk |
| Bayraktar et al. | Low risk | Low risk | Low risk | Low risk | Low risk | Low risk | Low risk |
| Zhu et al. | Low risk | Low risk | Low risk | Low risk | Low risk | Low risk | Unclear |
| Łukasz Zadrozny et al. | Low risk | Low risk | Low risk | Low risk | High risk | Low risk | High risk |
| Shihao Li et al. | Low risk | Low risk | Low risk | High risk | Low risk | Low risk | Low risk |
